# Residents or Tourists: Is the Lactating Mammary Gland Colonized by Residential Microbiota?

**DOI:** 10.3390/microorganisms12051009

**Published:** 2024-05-17

**Authors:** Ruomei Xu, Grace McLoughlin, Mark Nicol, Donna Geddes, Lisa Stinson

**Affiliations:** 1 School of Molecular Sciences, The University of Western Australia, Perth, WA 6009, Australiadonna.geddes@uwa.edu.au (D.G.); 2 School of Biomedical Sciences, The University of Western Australia, Perth, WA 6009, Australia; grace.mcloughlin@research.uwa.edu.au (G.M.); mark.nicol@uwa.edu.au (M.N.)

**Keywords:** human milk, microbiome, colonization, host–microbe interactions, bacteria

## Abstract

The existence of the human milk microbiome has been widely recognized for almost two decades, with many studies examining its composition and relationship to maternal and infant health. However, the richness and viability of the human milk microbiota is surprisingly low. Given that the lactating mammary gland houses a warm and nutrient-rich environment and is in contact with the external environment, it may be expected that the lactating mammary gland would contain a high biomass microbiome. This discrepancy raises the question of whether the bacteria in milk come from true microbial colonization in the mammary gland (“residents”) or are merely the result of constant influx from other bacterial sources (“tourists”). By drawing together data from animal, in vitro, and human studies, this review will examine the question of whether the lactating mammary gland is colonized by a residential microbiome.

## 1. Introduction

While human milk was initially assumed to be a sterile fluid, it is now widely accepted as a source of beneficial bacteria for infants, with around 10^4^ colony-forming units per mL [1]. The human milk microbiome exhibits a low richness of bacteria, with *Staphylococcus* and *Streptococcus* being the most abundant and prevalent genera [2,3,4,5,6]. The remaining portion is comprised of other typical skin and oral taxa, including *Corynebacterium* and *Cutibacterium* [7], as well as lactic acid-producing bacteria such as *Bifidobacterium* and *Lactobacillaceae*, which are considered to contribute to infant health via their production of immune-modulating metabolites [8]. Compared with formula-fed infants, breastfed infants have a lower incidence and severity of diarrhea, upper and lower respiratory tract infections, and otitis media [9]. Evidence also suggests that breastfeeding can positively affect children’s long-term health by reducing the risk of non-communicable diseases such as diabetes mellitus (type one and type two), obesity, childhood cancers, and cardiovascular diseases [10]. Given that aberrations to the human microbiota underpin a number of non-communicable diseases [11], the human milk microbiome has been identified as a potential mediator by which breastfeeding promotes infant health.

While much research has focused on characterizing the composition of the human milk microbiota, a fundamental question remains unanswered: do the bacteria recovered from milk actually colonize the lactating mammary gland (“residents”), or are they merely “tourists”, transiently present in milk? Given that human milk is a nutrient-rich environment, it might be expected that milk would contain a high load of bacteria; however, the biomass of the human milk microbiome is very low [12]. Further, recent data suggest that the viability and richness of the human milk microbiota are far less than previously expected, with less than half of the taxa detected using metataxonomic methods shown to be viable [13]. While the function of the human milk microbiota is often purported to be the transfer of microbes from mothers to infants, only a small portion of the milk microbiota actually colonize the infant gut despite constant exposure to milk during the breastfeeding period [14,15]. Collectively, these facts raise the question of whether these bacteria are “residents” or merely “tourists”, which are transiently present in the mammary gland.

Just as stool is a useful, though imperfect proxy for the gut microbiota, human milk may be an imperfect proxy for the lactating mammary gland microbiota. In order to better understand the local microbial ecology of the lactating mammary gland, biopsies are required. While such biopsies have been collected from non-lactating mammary glands [16,17,18], samples from lactating women are extremely difficult to obtain. Therefore, in this review, we draw together data from animal, in vitro, and human studies to examine the question of whether there is a residential microbiome in the lactating mammary gland. Such basic knowledge is foundational for our understanding of milk and early-life microbiome dynamics.

## 2. Conceptualizing a Residential Mammary Microbiome

While a residential microbiome is frequently assumed to be present in the lactating mammary gland, it is difficult to conceptualize the nature of this community. Unlike mucosal microbiome sites throughout the human body, such as the oral, vaginal, or gastrointestinal microbiome, there is no clear evidence that the lactating mammary epithelium is coated by a mucosal layer [19]. Therefore, bacterial adherence to the mammary epithelial cells must occur via another mechanism. The lactating mammary gland is constantly flushed, with any non-adhering cells removed during milk ejection. In this manner, the mammary microbiome may be similar to that of the urinary tract, in which bacteria with certain adherence factors are able to attach to the uroepithelium via specific binding to different glycoconjugate receptors [20]. However, unlike the urinary tract epithelium, mammary epithelial cells are secretory, constantly releasing lipids that act as decoy adherence sites for bacterial attachment [21]. Additionally, milk contains high levels of host antimicrobial proteins [22]. Therefore, in order to withstand flushing, secretion, and host defense mechanisms, mammary “residents” may be required to form biofilms with stratified structures. Bacteria become more resistant to harsh environments when a global infrastructure is assembled, and the extracellular matrix secreted by the bacteria protects them from antimicrobial molecules and from being removed by external forces [23]. While biofilm formation has been repeatedly noted in cases of mastitis or blocked ducts [24], it is unclear whether such structures are present in healthy mammary glands. The residential microbiota may or may not form prior to the onset of the first lactation and may become a reservoir of bacteria contributing to the human milk microbiome when the mammary gland is lactating. In addition to tourists or residents, there is a third option that may describe the nature of the milk microbiota. Given incomplete emptying of the mammary gland, bacteria remaining in the residual milk after each feed may provide inoculum for further bacterial growth, analogous to repeated-cycle batch fermentation.

## 3. Evidence That the Human Milk Microbiota Are “Tourists”

### 3.1. There Is a Constant Low-Grade Influx of Bacteria from other Sources

It is possible that the microorganisms detected in human milk do not actually colonize the mammary gland and instead reflect a constant influx of bacterial “tourists” from other sources. To date, there is a lack of experimental evidence demonstrating bacterial adhesion to the mammary epithelium. Instead, Stinson et al. suggested a constant influx model, in which bacteria constantly migrated into the lactating mammary gland, only to be washed out by milk ejection shortly thereafter [19]. The constant influx model and the lobular anatomy of the mammary gland, where each lobe is drained by its own duct through the nipple, may explain the low-grade variability in the composition of the human milk microbiota over the course of lactation as well as the low biomass of bacteria detected in milk [2,25]. Below, potential sources of influx are described.

#### 3.1.1. Infant Oral Cavity

During breastfeeding, nipple diameter increases as the infant applies a vacuum to remove the milk via openings of pores on the nipple surface, providing an entry point for infant oral bacteria [26]. As oxytocin degrades and the alveoli relax, milk is able to flow back into the gland providing transport for bacteria back into the lobes [26]. Indeed, the human milk microbiome has a high relative abundance of typical oral bacteria [2]. As such, the mode of feeding (bottle-feeding expressed milk versus direct breastfeeding) may be associated with different levels of introduction of oral flora to human milk. There is evidence that the milk from mothers who indirectly breastfeed their infants has lower bacterial richness as well as lower abundances of members of *Veillonellaceae* family, which could be the result of not being exposed to the infant’s oral microbiome as often [6]. Additionally, breastfeeding patterns, including breastfeeding frequency, have been associated with the composition and diversity of the milk microbiome [27]. These findings suggest that the composition of the milk microbiome is influenced by the constant influx of “tourists” from the infant oral cavity.

#### 3.1.2. Maternal Skin

Typical skin bacteria, including *Staphylococcus*, *Cutibacterium*, and *Corynebacterium*, are highly abundant in the human milk microbiome, suggesting a potential influx of bacteria from the epidermis of the breast and the nipple [2]. The presence of typical skin bacteria in breast biopsies taken from non-lactating women suggests that skin bacteria may be present in the breast prior to the onset of lactation [18]. In further support of this theory, a comparison of the human milk microbiome with that of non-human primate milk showed that human milk had lower bacterial richness [28]. This could be due to the relatively low abundance of bacteria on human skin, which results from human hygiene and cultural practices [28]. However, to date, no direct strain level evidence has demonstrated the influx of bacteria from the skin to the lactating mammary gland.

#### 3.1.3. Maternal Gut

The entero-mammary pathway has also been postulated as a constant source of bacterial “tourists” in human milk [29]. This theorized pathway may explain the presence of strictly anaerobic bacteria, such as *Bifidobacteria*, in human milk. In mice, gut lactic acid bacteria have been demonstrated to migrate via the lymphatic system to the mammary glands during pregnancy, and immune cells, including dendritic cells and macrophages, may be involved in this process [30]. These findings are in line with studies in which lactating women were administered oral *Lactobacillus*/*Ligilactobacillus* probiotics. The probiotics were able to be isolated from the milk samples of 6/10 of the treated mothers and 0/10 of the placebo mothers [31]. While further work is required to validate this putative pathway, current evidence supports the theory that a small percent of the milk microbiota originate from the maternal gut.

### 3.2. Human Milk Is a Hostile Environment for Bacteria

**Figure 1:**
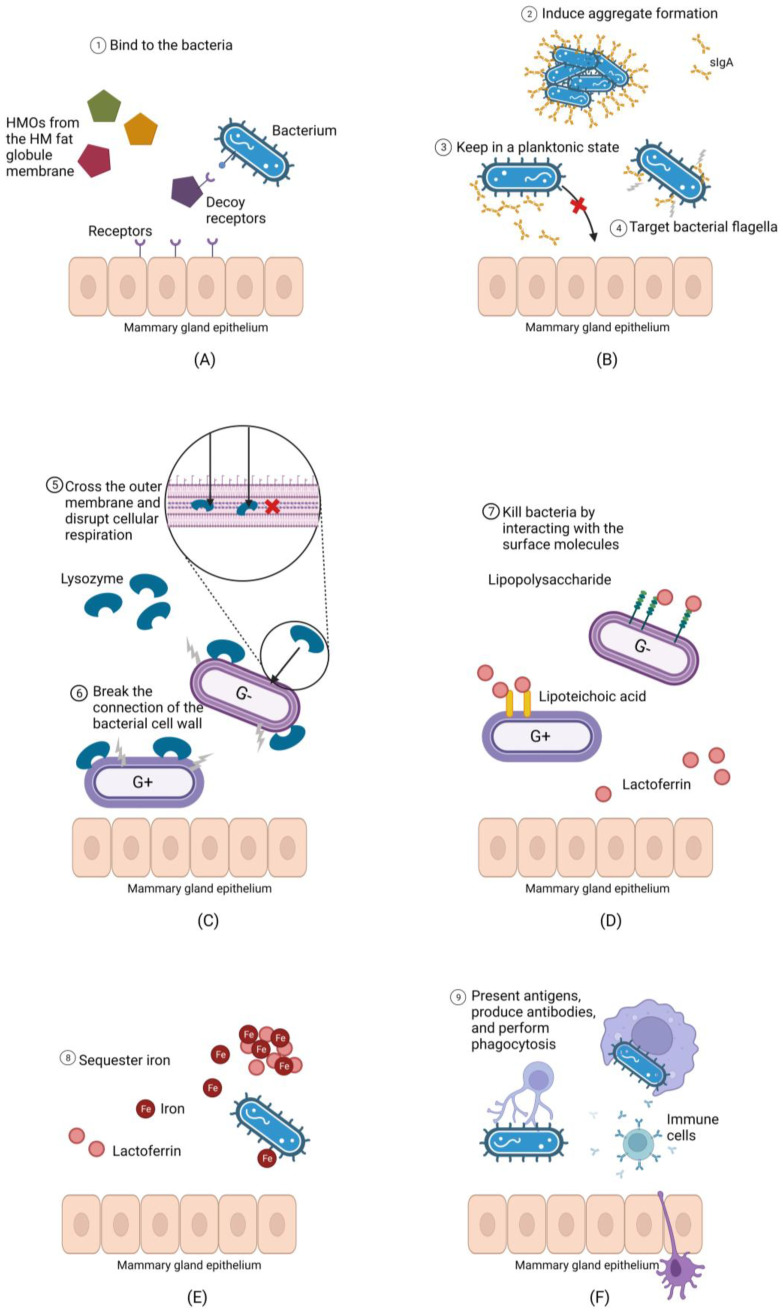
Human milk contains antimicrobial and anti-adhesive compounds that may prevent bacteria from colonizing the mammary gland. (**A**): Human milk oligosaccharides (HMOs) can bind bacteria, preventing them from adhering to the mammary epithelium, and increasing the likelihood that they will be flushed during milk ejection. (**B**): Secretory immunoglobulin A (sIgA) have multiple mechanisms through which they may prevent bacterial adherence to the mammary epithelium. (**C**): Lysozyme breaks linkages in the peptidoglycan cell wall of Gram-positive bacteria and can disrupt cellular respiration in both Gram positive and negative bacteria if it passes the cell wall. (**D**): Lactoferrin disrupts the Gram-negative bacterial cell wall via its interaction with lipopolysaccharide and compromises the Gram-positive cell wall via its binding to lipoteichoic acid, allowing other molecules such as lysozyme to penetrate. (**E**): Lactoferrin sequesters environmental iron required for bacterial growth. (**F**): Host immune cells present in human milk can produce antibodies against milk bacteria and perform phagocytosis.

Once bacteria have entered the mammary gland, they face challenges to colonization due to the antimicrobial compounds in milk, including secretory immunoglobulin A (sIgA), human milk oligosaccharides (HMOs), lysozyme, lactoferrin, and immune cells. Human milk plays an important role in defending infants from infection and initially evolved for this purpose [32]. The distinctly antimicrobial nature of milk means that milk is theoretically a hostile environment for bacteria (Figure 1). As bacteria enter the mammary gland via constant influx from other sources, they may fail to endure suppression by antimicrobial and anti-adhesive compounds in human milk, which may explain the low bacterial load in this biofluid.

#### 3.2.1. Secretory Immunoglobulin A

As the major mucosal antibody in the human body, sIgA contributes to microbiome homeostasis by modulating the growth of commensal and potentially pathogenic bacteria [33,34]. It has been demonstrated by previous studies that sIgA plays a key role in innate immunity through several mechanisms, including targeting the flagella on the surface of microorganisms to reduce their motility [35], binding dividing bacteria and inducing them to form large aggregates for immune exclusion [36], and hydrophilic entrapment of microorganisms [37]. Thus, sIgA may prevent the adherence of bacteria to the mammary epithelium, keeping bacterial cells in a planktonic state that increases their likelihood of being washed away by milk ejections stimulated during breastfeeding [26].

Interestingly, 40% of human milk bacteria are bound to sIgA [38]. This binding likely facilitates the safe transmission of maternal bacteria to infants during breastfeeding, with breastfed infants harboring significantly higher levels of sIgA-coated bacteria in their stool than formula-fed infants [39]. Additionally, the levels of sIgA-bound milk bacteria are significantly higher than in other body sites, with approximately 11% of fecal bacteria and 17% of salivary bacteria bound to sIgA [40]. Further, levels of sIgA-coated bacteria rise to >60% in the gut during inflammatory processes [41]. This observation aligns with the theory that lactation evolved from inflammatory responses [32]. Due to the high level of sIgA-coated bacteria and the bacterial clearance functions of this antibody, sIgA may prevent the attachment of bacteria to the mammary gland and aid in their removal. If bacteria in milk are not able to overcome immune exclusion, attach to the epithelium, or form biofilms, they may be unable to colonize the lactating mammary gland.

#### 3.2.2. Human Milk Oligosaccharides

HMOs are the third most abundant component in human milk [42]. The high abundance of HMOs in milk despite the inability of infants to fully digest them, suggests an important role for HMOs; otherwise, they would have been lost during the evolutionary process. While it is well-recognized that HMOs play a prebiotic, and particularly bifidogenic, role in the infant gut, they also have antibacterial properties. HMOs can inhibit the adhesion of pathogens by acting as decoy receptors that bind harmful bacteria, including *Pseudomonas aeruginosa*, *Salmonella enterica*, and enteropathogenic *Escherichia coli* [43]. One study found that HMOs isolated from milk samples of nine healthy participants could suppress the growth of *Staphylococcus aureus*, *Enterococcus faecium*, and *Enterococcus faecalis*, regardless of whether the bacteria were planktonic or in biofilms [44]. HMOs also have the ability to enhance cytokine secretion in vitro [45]. Therefore, given their antibiofilm, anti-adhesive, and cytokine-enhancing properties, HMOs in milk may make colonization of the lactating mammary gland difficult.

#### 3.2.3. Lysozyme and Lactoferrin

Human milk contains two major antimicrobial proteins: lysozyme and lactoferrin [22]. Lysozyme is a bacteriolytic enzyme that can lyse bacteria by breaking the connection between N-acetylglucosamine and N-acetylmuramylpentapeptide of the Gram-positive bacterial cell wall [46]. This enzyme can also kill Gram-negative bacteria by crossing the outer membrane and disrupting cellular respiration [47]. Lactoferrin is a glycoprotein found in most mammalian milk, with human milk having the highest concentration [48]. The antibacterial activity of lactoferrin is partially due to its highly cationic N-terminal region [49]. This region destroys bacteria by interacting with lipoteichoic acid in Gram-positive bacteria and lipopolysaccharide in Gram-negative bacteria [49]. Lactoferrin can also repress the growth of bacteria by sequestering iron, which is an essential micronutrient for many microorganisms [50]. Further, lactoferrin can disrupt the motility of bacteria by binding to bacterial flagella and prevent the formation of biofilms by interacting with cellular determinants that are essential for adherence [51].

There is increasing evidence that lactoferrin and lysozyme can act synergistically, increasing their antibacterial properties, particularly for Gram-negative microorganisms because lactoferrin can alter the permeability of the outer membrane, which provides lysozyme with better access to the cell wall peptidoglycan [52]. This may explain why the majority of milk microbiota are Gram-positive. In addition, it has been demonstrated that lactoferrin and lysozyme in combination can lead to enhanced bacterial clearance of *Staphylococcus epidermidis* in vitro [53]. Although the antibacterial ability of the two molecules might be strain-specific, the powerful antimicrobial properties of lysozyme and lactoferrin are likely to contribute to the low viability of bacteria in human milk.

#### 3.2.4. Immune Cells

Apart from the range of antimicrobial molecules outlined above, human milk also contains leukocytes, including macrophages, lymphocytes, and monocytes [54]. Immune cell populations vary temporally in milk, with leucocytes constituting up to 70% of the cells in colostrum and decreasing to 2% in mature human milk [55]. This is likely due to the open tight junctions of the mammary gland epithelium in the first few days following birth, allowing paracellular transport of immune cells into the mammary gland [56]. The high level of leucocytes in colostrum may also reflect the need for immunological protection during the newborn period.

It is possible that these activated and motile immune cells may suppress the growth of milk bacteria. A high potion of milk bacteria (66% in colostrum and 36% in mature milk) are reported to be associated with immune cells, suggesting extensive interaction between milk microbes and host immune cells [57]. Bacteria aggregated to immune cells are unlikely to actively replicate or colonize [57]. Although the presence of immune cells in milk is not always associated with infections, the number of human milk immune cells increases significantly in lactating women with mastitis, suggesting a strong inflammatory response to the overgrowth of bacteria in the mammary gland [55]. Interestingly, there is an increase in human milk immune cells in response to active infections in a nursing infant [58]. It is speculated that a local immune response could be triggered when bacteria from the oral cavity of ill infants enter the mammary gland via a retrograde flow of milk during breastfeeding [26]. In addition, it has been found that the milk from exclusively breastfeeding mothers has a larger number of immune cells compared with non-exclusive breastfeeding mothers, suggesting that leukocyte responses are simulated by frequent exposure to microorganisms from the infant’s oral cavity [55]. Collectively, these data suggest that increased or aberrant bacterial exposure in the lactating mammary gland triggers an influx of maternal immune cells, which may suppress bacterial growth and prevent bacterial colonization.

## 4. Evidence That the Human Milk Microbiota Are “Residents”

### 4.1. Human Milk Is a Nutrient-Rich Environment

Similar to other colonized body sites, such as the oral cavity and gastrointestinal tract, the mammary gland is a nutrient-rich environment that may promote the growth of microorganisms. Human milk contains a high abundance of macronutrients: proteins, sugars, and lipids [59]. The composition of human milk macronutrients changes over the lactation period, with higher protein and lower lactose in colostrum compared with mature milk [60].

As the main carbon source in milk, lactose likely acts as an energy source of several dominant milk taxa including *Staphylococcus*, *Streptococcus*, and lactic acid bacteria (LAB) [61]. These bacteria metabolize lactose and produce galactose and then subsequently lactate, short-chain fatty acids, and gases by their β-galactosidase activity [62]. Most bacteria in human milk are lactose-fermenting, suggesting adaptation to the lactose-rich environment in human milk. This is strongly suggestive of a residential microbiome. However, to date, there is no experimental evidence that demonstrates that the bacteria in human milk metabolize lactose in situ. While the by-products of lactose metabolism, such as lactate and short-chain fatty acids, are found in human milk, these may be derived from maternal circulation. Further work is therefore needed to clarify the metabolic activity of bacteria within the lactating mammary gland.

Apart from carbohydrates, lipids are also important nutrients in human milk and provide infants with more than half of their caloric intake [63]. Bacteria, including LAB and *Bifidobacteria*, can metabolize lipids [64]. Furthermore, lipids may provide the milk bacterial communities with advantages by membrane trafficking and evading host immune exclusion [65].

Collectively, human milk contains many macro- and micronutrients that may support the growth of bacteria. Being a warm, wet, and nutrient-rich environment, it would be unusual for bacteria not to colonize the lactating mammary gland to some degree despite the highly antibacterial nature of the fluid.

### 4.2. Biofilm Formation

Microorganisms are able to colonize and persist within host environments using many strategies, including biofilm formation [66]. Biofilms may allow bacteria to persist in the lactating mammary gland in spite of the presence of antibacterial molecules and constant flushing of human milk. There is evidence (outlined below) that many bacteria detected in human milk may form biofilms (Table 1), which increases the possibility that these microorganisms are colonizers of the lactating mammary gland. Biofilm formation is part of the etiology of mastitis; however, there is currently no evidence of the presence of biofilms in healthy lactating mammary glands. Notably, if bacteria do utilize biofilm formation to colonize the mammary glands, this may explain the low titer of bacteria in human milk, as the bacteria would be largely protected from being washed out with milk ejections.

#### 4.2.1. *Staphylococcus* Species

Staphylococci are widely prevalent and highly abundant in human milk. Despite often being associated with human mastitis, *S. aureus* is present in 6–45% of human milk samples collected from healthy lactating women [67,68,69,70]. The factors and biological contexts that convey virulence to this species in milk are currently unclear; however, there is evidence that the growth of *S. aureus* may suppressed by certain commensal bacteria in human milk [71]. There are several mechanisms that would allow *S. aureus* to colonize the lactating mammary gland. For instance, genes including *eno*, *icaA*, and *icaD* are associated with the biofilm-forming ability of this microorganism [72].

Apart from *S. aureus*, coagulase-negative staphylococci can also form biofilms. *S. epidermidis*, one of the most common coagulase-negative *Staphylococcus* species in human milk, may adhere to the mammary epithelial cells with the help of laminin-binding protein encoded by the *eno* gene [73]. Notably, *S. epidermidis* and *S. aureus* are ubiquitously present on human skin [74] and are also found within the infant’s oral cavity [2] and may, therefore, enter the mammary gland via nipple openings during breastfeeding.

#### 4.2.2. *Streptococcus* Species

*Streptococcus* species such as *Streptococcus salivarius*, *Streptococcus mitis*, *Streptococcus anginosus*, and *Streptococcus parasanguinis* are frequently detected in the human milk microbiome [2].

The biofilm-forming ability of *S. salivarius* has been demonstrated in vitro, with genes encoding the CshA surface-exposed protein and GtfH glycosyltransferase contributing to the auto-aggregation and biofilm formation of *S. salivarius* [75]. Other genes such as *Asp1*, *CwpB*, *GtfG*, *SecA2*, and *SrtA* are also considered to play a role in *S. salivarius* adhesion to HT-29 human epithelial cell lines (colon adenocarcinoma; ATCC HTB-38) [75]. *S. salivarius* can inhibit biofilm formation from other bacteria including *S. mutans* [76], which increases the likelihood of *S. salivarius* securing a niche in the mammary gland.

Similar to *S. salivarius*, *S. mitis* contains various genes that facilitate host-cell adhesion, including *cdaA*, *pde1*, and *pde2* [77]. A study investigating 12 strains of *S. mitis* isolated from the oral cavity of healthy infants provided evidence of the biofilm-forming abilities of these microorganisms in vitro [78]. Because the mammary gland is constantly exposed to the infant’s oral microbiome during breastfeeding [26], it is likely that *S. mitis*, which can form biofilms in the infant’s oral cavity, has the ability to colonize and adhere to the mammary gland epithelium. However, the milk ducts do not exhibit evidence of a mucous membrane, suggesting that different adhesion mechanisms may be necessary.

The biofilm formation of *S. anginosus* relies primarily on the *luxS* gene, and bacteria carrying this gene are more likely to be resistant to antimicrobial compounds [79]. The ability of *S. parasanguinis* ability to attach to host cells depends on fimbriae-associated protein 1, which is an important adhesin found in many streptococci [80]. Furthermore, a recently identified protein, BapA1, on the surface of *S. parasanguinis*, plays a role in biofilm formation, with BapA1-deficient mutants unable to auto-aggregate and form biofilms in vitro [81]. The biofilm-forming ability of these species may allow them to survive the harsh milk microenvironment and colonize the lactating milk ducts [82].

#### 4.2.3. *Enterococcus* Species

*Enterococcus* species isolated from human milk harbor bacteriocins that inhibit the growth of neonatal pathogens [83]. Research has demonstrated that *E. faecalis* isolated from bovine milk has the ability to adhere to bovine mammary epithelial cells, an ability that is enhanced in the presence of bovine milk [84]. These findings suggest that milk components play a role in the biofilm formation of this microorganism. Similarly, the formation of *E. faecium* biofilms is enhanced by neutral pH and a nutrient-rich environment, which are available in human milk [85].

#### 4.2.4. Lactic Acid Bacteria

Although LAB such as *Lactobacillaceae* have a relatively low abundance in human milk, there is a possibility that they form biofilms. In a study of the biofilm-forming abilities of *Lactococcus* and *Lactobacillaceae* in bovine mammary epithelial cells, all 13 tested LAB strains were observed to adhere to the mammary epithelial cells, although the mean adhesion percentage varied between strains (30–68%) [86]. Interestingly, *Lactobacillaceae* species are the main contributors to bacterial biofilm architecture in the human oral cavity [87], suggesting that biofilm-forming LAB may enter and subsequently colonize the lactating mammary gland.

**Table 1:** Evidence for the biofilm-forming potential of bacteria previously detected in human milk.

	Biofilm-Forming Bacteria	Reference	Origins of Tested Isolates	Number of Tested Strains	Biofilm Testing Method	Biofilm Genes or Proteins Identified	Study Findings
*Staphylococcus* species	*S. aureus* and coagulase-negative *Staphylococcus*	Darwish and Asfour 2013 [72]	Bovine mastitis milk samples	108 (40 *S. aureus* and 68 coagulase-negative *Staphylococcus* isolates)	Congo Red Agar method; microtiter plate method	Genes *eno*, *icaA*, *icaD*, and *bap*	By the Congo Red Agar method, 67.5% of *S. aureus* and 72.1% of coagulase-negative *Staphylococci* were biofilm producers; by the microtiter plate method, 100% of *S. aureus* and 94.1% of coagulase-negative *Staphylococci* were biofilm producers.
	Coagulase-negative *Staphylococcus* including *S. chromogenes*, *S. simulans*, and *S. epidermidis*	Simojoki et al., 2012 [73]	Bovine mastitis milk samples	244 isolates	Tissue culture plate assay and fluorescent in situ hybridization	Genes encoding the adhesion proteins MSCRAMM, as well as biofilm-associated proteins eno and bap	A total of 40% of tested *S. epidermidis* isolates produced slime.
*Streptococcus* species	*S. mitis*	Rørvik et al., 2021 [77]	Mutants generated by markerless gene editing	8 strains	0.1% Safranin staining and quantification	Genes *cdaA*, *pde1*, and *pde2*	*S. mitis* biofilm formation was associated with gene *cdaA*, *pde1*, and *pde2*.
	*S. parasanguinis*	Chen et al., 2020 [88]	Type cultures	6 strains	Biofilm structure was examined by confocal laser scanning microscopy	Protein Fap1, BapA1, and FimA	*S. parasanguinis* biofilm formation was associated with collagen-binding proteins.
	*S. mitis*	Harth-Chu et al., 2019 [78]	Oral mucosal sites of healthy infants; oral cavity, dental biofilms, or bloodstream of patients with clinical symptoms of bacteremia or septicemia	20 strains	Microtiter plate method	Genes *pcsB*	*S. mitis* biofilm formation was associated with gene *pcsB*.
	*S. anginosus*	Perez-Tanoira et al., 2019 [82]	Salivary stones from patients with sialolithiasis	10 isolates	Fluorescence microscopy and sonication	··	*S. anginosus* had the ability to form biofilms in the human oral cavity.
	*S. salivarius*	Couvigny et al., 2018 [75]	Wild-type strains and mutants, clinical isolates from human blood, oral cavity, human milk, sputum, peritoneal cavity, trachea, and lower-left lung	28 isolates	Confocal laser scanning microscopy	Gene *bglB*, *cshA*, *gtfH*, *liaR*, *asp1*, *asp2*, *cwpB*, *cwpK*, *gtfE*, *gtfG*, *secA2*, and *srtA*	*S. salivarius* had the ability to auto-aggregate and form biofilms.
	*S. parasanguinis*	Liang et al., 2011 [81]	Type cultures	7 strains	Microtiter plate method and plastic coverslip method	Cell surface protein BapA1	Protein BapA1 contributed to the biofilm formation of *S. parasanguinis*.
*Enterococcus* species	*E. faecium*	Maurya et al., 2021 [85]	Sludge samples	1 isolate	Tube assay method: biofilms were measured based on optical density	··	*E. faecium* biofilm formation was significantly influenced by physiological conditions.
	*E. faecalis*	Elhadidy and Zahran, 2014 [84]	Bovine mastitis milk samples	3 strains	Semi-quantitative adherence assay: biofilms were measured based on optical density	··	Growth in milk enhanced biofilm formation of *E. faecalis*.
Lactic acid bacteria	*Lb. rhamnosus*, *Lactococcus. lactis* subsp. *Lactis*, *Lb. plantarum*, *Lb. paracasei* subsp. *Paracasei*, *Lb. brevis*, and *Lb. buchneri*	Wallis, Krömker and Paduch, 2018 [86]	American Type Culture Collection; bovine milk samples; animal bedding samples	13 strains	Optical density	··	Biofilm formation was observed in all tested strains.
	*Lactococcus* spp. and *Lactobacillus* sp.	Zijnge et al., 2010 [87]	Human dental plaque	Not described	Fluorescent in situ hybridization	··	*Lactobacillus sp*. was the main contributor to human oral microbial biofilms.

### 4.3. Bacterial Detection in Non-Lactating Mammary Glands and in Pre-Colostrum

If the milk microbiota represents “residents”, it might be expected that a microbiome is present in the mammary gland outside of the context of lactation. A metataxonomic study of nipple aspirate fluid identified bacterial DNA in healthy women who were not lactating or had lactated within the previous year. While many of the recovered taxa appear to represent reagent-derived contamination (e.g., *Sphingomonadaceae*), typical milk taxa were also detected [89]. If the non-lactating human mammary gland houses a microbiome, the advent of lactation would introduce new substrates for bacterial metabolism, as well as new antimicrobial conditions (such as antimicrobial proteins, immune cells, etc.), and new bacterial exposures (such as the infant oral cavity). These experiences may alter the composition or load of bacteria in the breast during the course of lactation. It would, therefore, be of interest to study the microbiome of non-lactating mammary glands before and after the first lactation, as well as the relationship between the human milk microbiome and the bacterial profiles of the breast tissue itself. However, such studies rely on invasive sampling and are therefore limited. Additionally, there is evidence that milk secretions produced prior to delivery and commencement of breastfeeding (“pre-colostrum”) harbor a microbiota that is very similar to that of mature milk [90]. In a small study of 20 mothers, 15 of which were primiparous, numerous typical human milk and infant oral taxa were detected using both culture-dependant and culture-independent techniques in samples collected at 38–40 weeks gestation [90]. The presence of such taxa prior to contact with the infant suggests that the bacteria detected in milk are not purely contaminants from the infant’s oral cavity. Indeed, the open tight junctions between the mammary epithelial cells prior to the onset of full lactation may aid in internal seeding of the milk microbiome [91].

## 5. Evidence for the “Repeated-Cycle Batch Fermentation” Model

Given that the lactating mammary gland is never truly empty [92], bacteria will remain in the residual milk after each episode of milk expression or infant feeding. These bacteria may then replicate and act to seed the newly synthesized milk within the gland. Data demonstrating a highly stable microbiota in human milk across lactation support this theory [93,94]. Further, this model would not require adherence of bacteria to the mammary epithelium, as planktonic cells are also able to replicate. This model would, therefore, allow a transition from “tourists” to “residents”, whereby a consistent and permanent microbiota exists suspended in milk that experiences a large degree of emigration and potential immigration from exogenous sources.

## 6. Knowledge Gaps

Human milk is a hostile environment with high levels of antimicrobial compounds such as HMOs, sIgA, lysozyme, lactoferrin, and immune cells. It may be difficult for bacteria to overcome immune exclusion, attach to the mammary epithelium, and form biofilms, which are necessary steps for colonization. However, human milk is also a nutrient-rich biofluid constantly exposed to external and internal bacterial sources. Whether biofilm formation occurs in healthy lactating breasts remains unknown.

In order to better understand the nature of the milk microbiome, studies investigating biofilm markers in milk from healthy mothers or in vitro mammary epithelial cell culture studies are required. Conclusive evidence on the existence of a residential microbiome in the lactating human mammary gland may be obtained via tissue biopsies from lactating women; however, such samples are difficult to obtain.

High-quality strain-level evidence is required to assess the extent of bacterial sharing between the lactating mammary gland and exogenous sources (such as the infant’s oral cavity and maternal skin). There is likely a bidirectional nature to these relationships; however, the extent of bacterial transfer in each direction is currently unclear. Data on the consistency of the milk microbiota between subsequent lactations would also be highly valuable in assessing the possibility of a consistent residential microbiome. While there is some evidence suggesting that the milk microbiome is highly stable across lactation [93,94], species- or strain-level studies are needed to confirm these findings.

## 7. Weighing the Evidence

In this review, we have synthesized the data in support of each side of the argument. Collectively, the evidence suggests that bacteria resembling the mature milk microbiota are present in the mammary gland prior to delivery of the infant and that this initial community is bolstered and shaped by subsequent contact with the infant’s oral cavity and other exogenous exposures. However, given evidence that milk is a hostile environment for bacteria, these bacteria may behave as “tourists”, which are transiently present in the lactating mammary gland before being ejected via milk ejections. Their adherence to mammary epithelial cells is unlikely, given the myriad of antibacterial and anti-adherence molecules in milk. Nevertheless, bacteria that survive each milk ejection may go on to inoculate the incoming synthesized milk, acting as a repeated-cycle batch fermentation. This theory helps to explain the low biomass, low diversity, and low viability of the human milk microbiome. Collectively, it is very likely that all three models contribute to the milk microbiome to different extents. Thus, future research in the field should focus on the possible contribution of each mechanism, particularly in regard to host and physiological factors, lactation stage, feeding mode and frequency, and breastfeeding history. This fundamental question impacts our interpretation of data in this field. More clarity in this area may aid in future attempts to understand the etiology of mastitis and the role of the milk microbiota in infant health.

## Data Availability

Not applicable.
